# Idiopathic Left Anterior Fascicular Ventricular Tachycardia in the Emergency Department: A Case Report

**DOI:** 10.5811/westjem.65063

**Published:** 2026-09-26

**Authors:** Rochelle Kofman, Connor O’Hare, Lauren B. Querin

**Affiliations:** * Mayo Clinic Alix School of Medicine, Scottsdale, Arizona; † Mayo Clinic Hospital Phoenix, Department of Emergency Medicine, Phoenix, Arizona

**Keywords:** idiopathic fascicular ventricular tachycardia, tachyarrhythmia, verapamil, case report

## Abstract

**Introduction:**

Idiopathic fascicular ventricular tachycardia is a rare form of ventricular tachycardia that is commonly seen in young, otherwise healthy individuals without structural heart disease. This rhythm can be difficult to distinguish from other forms of tachyarrhythmia, including supraventricular tachycardia, yet acute pharmacologic management of this condition varies significantly from more common forms.

**Case Report:**

We describe a 42-year-old woman with no cardiac history who presented with palpitations and lightheadedness. She was found to be in a hemodynamically stable, wide-complex tachycardia. Given no prior cardiac history, supraventricular tachycardia with aberrancy was initially considered as a leading diagnosis; however, a trial of adenosine and lidocaine was ineffective. Her electrocardiogram revealed a regular wide complex tachycardia at 262 beats per minute with a right bundle branch block morphology and right axis deviation, consistent with a rare left anterior fascicular subtype of ventricular tachycardia. She later underwent successful ablation of the left anterior fascicle with no recurrence of symptoms at follow-up.

**Conclusion:**

This report highlights a case of idiopathic fascicular ventricular tachycardia successfully treated with intravenous verapamil and left anterior fascicle ablation, addressing the diagnostic challenges of this arrhythmia. Early recognition by emergency physicians is essential to avoid treatment delays and ensure appropriate definitive management.

## INTRODUCTION

Ventricular tachycardia (VT) is a high-risk cardiac arrhythmia that most commonly occurs in the setting of structural heart disease and myocardial scar. However, approximately 10% of patients are young and otherwise healthy with no known cardiac disease.^1^–^3^ These cases are referred to as idiopathic fascicular VT and often stem from re-entrant pathways within the His-Purkinje system.^3^ The large majority of these pathways originate from the left posterior fascicle, while the left anterior fascicular subtype represents only 5–10% of idiopathic fascicular VT cases.^4^,^5^ While idiopathic fascicular VT generally has a better prognosis than other forms of VT, it is often difficult to recognize on initial presentation. This diagnostic challenge in the emergency department (ED) arises because its clinical and electrocardiographic features often mimic other arrhythmias such as supraventricular tachycardia (SVT).^6^,^7^ In addition to its diagnostic challenge, idiopathic fascicular VT characteristically responds to verapamil, an agent not commonly used and potentially even contraindicated in other forms of arrhythmia^8^. It is vital that emergency physicians recognize and differentiate idiopathic fascicular VT from other arrhythmias.

## CASE REPORT

A 42-year-old woman presented to the ED with palpitations, tachycardia, lightheadedness, and nausea. She had no known past medical history and no personal or family history of heart disease or sudden cardiac death. She reported moderate alcohol intake the night prior, followed by multiple episodes of nonbloody emesis overnight. In the morning, she had another episode of emesis and began experiencing palpitations and lightheadedness. She denied syncope or chest pain. She stated that she has never had symptoms like this in the past and denied illicit drug use.

Upon arrival to the ED, she was tachycardic to 240 beats per minute (bpm) with a blood pressure of 144/123 mm Hg, while other vitals were within normal limits. She appeared fatigued and mildly anxious. Physical examination was notable for tachycardia and dry mucous membranes; she was alert, conversant, and nontoxic in appearance. Laboratory results ordered initially during the patient’s evaluation showed mild leukocytosis and mild transaminitis, while electrolytes, thyroid studies, pregnancy testing, and chest radiography were unremarkable.

The initial electrocardiogram (ECG) (Image) demonstrated a regular wide complex tachycardia at 262 bpm with a right bundle branch block-type morphology and right axis deviation. There were no clear capture beats or fusion complexes on the 12-lead ECG. Initial differential diagnoses were SVT with aberrancy versus VT.

*CPC-EM Capsule*

What do we already know about this clinical entity?

*Idiopathic fascicular ventricular tachycardia is a rare subtype that can resemble supraventricular tachycardia.*

What makes this presentation of disease reportable?

*This highlights a rare subtype, idiopathic anterior fascicular ventricular tachycardia, treated with intravenous verapamil and left anterior fascicle ablation.*

What is the major learning point?

*This arrhythmia may mimic more common tachyarrhythmias, but early recognition is essential to avoid treatment delays and ensure definitive management.*

How might this improve emergency medicine practice?

*Prompt identification by clinicians can prevent delays in care, adverse outcomes from inappropriate management, and facilitate definitive management.*

A trial of intravenous adenosine (6 mg) had no effect on the rhythm. Subsequent administration of intravenous lidocaine (50 mg bolus followed by infusion) resulted in partial rate reduction to the low 220 bpm but was discontinued due to mild hypotension and poor efficacy. Cardiology consult recommended a trial of verapamil (2.5 mg) intravenously, which resulted in conversion to sinus rhythm.

The patient was admitted to cardiology, and electrophysiology was consulted for mapping and ablation. An echocardiogram showed no structural heart disease with normal biventricular function. The patient later underwent empiric ablation of the left anterior fascicle with noninducible VT following the procedure. A follow-up ECG one month later was normal, and the patient had no recurrence of symptoms.

## DISCUSSION

Idiopathic fascicular VT represents approximately 10% of all presenting cases of VT and is due to a re-entrant circuit with ectopic focus most commonly within the left ventricle.^3^ This arrhythmia typically affects young, otherwise healthy individuals between the ages of 15–40 years, with a male predominance of 60–80%.^9^ It was first described in 1979 by Zipes et al and in 1981, Belhassen et al identified it as verapamil-sensitive VT.^1^,^10^ Like other forms of VT, idiopathic fascicular VT presents as a monomorphic wide complex tachycardia with fusion complexes, atrioventricular (AV) dissociation, and capture beats. The QRS complex duration, however, is usually only modestly widened (100–140 milliseconds) compared with other forms of VT.^11^,^12^ Other characteristic ECG findings include a right bundle branch block morphology and axis deviation which is dependent on the anatomical site of the re-entry circuit.^11^ The anatomic location of the majority of re-entry circuits is at the left posterior fascicle, which produces a left axis deviation on ECG. A right axis deviation, as in this patient’s case, is seen more rarely (5–10% of cases) when the re-entrant circuit is in the left anterior fascicle, making it a particularly rare clinical entity.^11^ Recognition of this variant is important, as its atypical axis may contribute to further uncertainty in the diagnosis of an already rare arrhythmia.

Clinical presentation is variable and may include dizziness, palpitations, chest pain, shortness of breath, or syncope. Given occurrence is primarily in young, healthy individuals, hemodynamic stability is more common than in other forms of VT.3 These clinical features, along with the rhythm’s relatively organized appearance, contribute to the diagnostic challenge of the arrhythmia because it mimics other tachyarrhythmias such as SVT with aberrancy.^11^,^12^ It is imperative that emergency physicians be aware of idiopathic fascicular VT and consider it in their differential of tachyarrhythmia since the acute pharmacologic management of this condition varies from other more common forms. The diagnosis can be made by carefully observing specific features of VT, including fusion complexes, capture beats, and AV dissociation, as well as characteristic right bundle branch block morphology and axis deviation.^11^

As seen in this case, idiopathic fascicular VT is often refractory to commonly used pharmacological agents for tachyarrhythmia. It may be unresponsive to adenosine, beta-blockers, and lidocaine; however, it does classically respond to intravenous verapamil, a nondihydropyridine (DHP) calcium channel blocker.^13^,^14^ Although not entirely understood, verapamil is thought to be effective as the re-entrant circuit is calcium channel dependent. Some studies have also demonstrated efficacy of other non-DHP calcium channel blockers such as diltiazem.14 It should also be noted that calcium channel blockers are not typically recommended in other forms of VT.15 However, like other forms of wide complex tachycardia, an unstable patient should be treated with synchronized cardioversion.^10^ In cases of diagnostic uncertainty, synchronized cardioversion should always remain a key consideration. Definitive management involves timely coordination with electrophysiology consultation for evaluation of catheter ablation to terminate the aberrant pathway.^10^

A key clinical consideration is the potential for initial misdiagnosis as SVT with aberrancy. As in such cases, adenosine is often administered as first-line therapy, including in this patient’s case.^11^,^12^ While generally safe, adenosine is frequently ineffective in fascicular VT and may delay appropriate treatment. Physicians should maintain a high index of suspicion for idiopathic fascicular VT in patients initially presumed to have SVT who do not respond to adenosine. Early recognition is important because verapamil may be both diagnostic and therapeutic in this arrhythmia.

This case highlights a rare presentation of idiopathic fascicular VT arising from the left anterior fascicle, an uncommon subtype that poses an even greater diagnostic challenge. Although most patients presenting with monomorphic VT have underlying heart disease, idiopathic fascicular VT is often seen in young, healthy individuals without structural heart disease. As demonstrated in this case, diagnosis can be challenging because the rhythm can mimic other forms of tachyarrhythmia, including SVT with aberrancy. Despite the diagnostic challenge, early identification of this rhythm is imperative because effective pharmacologic treatment varies from other forms of tachyarrhythmia, including other types of VT.^11^,^12^ Definitive management with targeted cardiac ablation techniques has been highly successful in preventing recurrence and achieving favorable long-term outcomes in idiopathic fascicular VT.^11^

## CONCLUSION

Idiopathic fascicular ventricular tachycardia often presents in young, otherwise healthy patients and can closely mimic more common rhythms such as SVT with aberrancy, increasing the risk of misdiagnosis. Unlike typical management of wide complex tachycardias, this arrhythmia is frequently unresponsive to adenosine and instead responds to verapamil, making early recognition critical for appropriate treatment. Prompt identification can prevent delays in care and facilitate timely cardiology consultation or referral for definitive management with catheter ablation.

## Figures and Tables

**Image:**
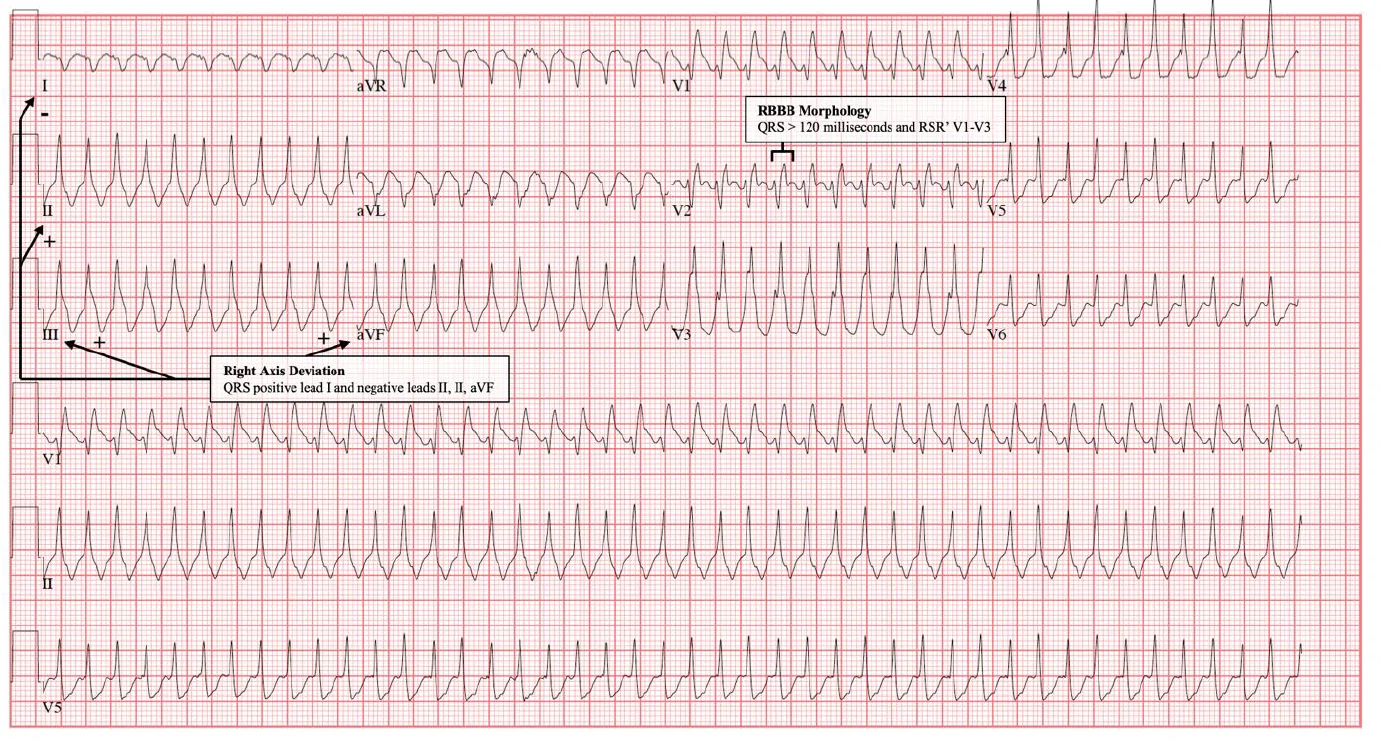
Initial electrocardiogram demonstrating a regular, wide complex tachycardia with a right bundle branch block morphology and a right axis deviation.

## References

[1] Zipes DP, Foster PR, Troup PJ (1979). Atrial induction of ventricular tachycardia: reentry versus triggered automaticity. Am J Cardiol.

[2] Ali H, Scheinman MM, Higuchi S (2024). Left fascicular ventricular tachycardia: What is forgotten in the differential diagnosis?. Circ Arrhythm Electrophysiol.

[3] Lerman BB, Stein KM, Markowitz SM (1997). Mechanisms of idiopathic left ventricular tachycardia. J Cardiovasc Electrophysiol.

[4] Kapa S, Gaba P, DeSimone CV (2017). Fascicular ventricular arrhythmias: pathophysiologic mechanisms, anatomical constructs, and advances in approaches to management. Circ Arrhythm Electrophysiol.

[5] Alahmad Y, Asaad NA, Arafa SO (2017). Idiopathic fascicular left ventricular tachycardia. Heart Views.

[6] Srivathsan K, Lester SJ, Appleton CP (2005). Ventricular tachycardia in the absence of structural heart disease. Indian Pacing Electrophysiol J.

[7] Chew HC, Lim SH (2007). Verapamil for ventricular tachycardia. Am J Emerg Med.

[8] Buxton AE, Marchlinski FE, Doherty JU (1987). Hazards of intravenous verapamil for sustained ventricular tachycardia. Am J Cardiol.

[9] Prystowsky EN, Padanilam BJ, Joshi S (2012). Ventricular arrhythmias in the absence of structural heart disease. J Am Coll Cardiol.

[10] Belhassen B, Rotmensch HH, Laniado S (1981). Response of recurrent sustained ventricular tachycardia to verapamil. Br Heart J.

[11] Francis J, Venugopal K, Khadar SA (2004). Idiopathic fascicular ventricular tachycardia. Indian Pacing Electrophysiol J.

[12] Chiu C, Sequeira IB (2004). Diagnosis and treatment of idiopathic ventricular tachycardia. AACN Clin Issues.

[13] Wang JC, Lim SH, Teo WS (2002). Calcium channel blockers as first line treatment for broad complex tachycardia with right bundle branch block: ingenuity or folly?. Resuscitation.

[14] Gill JS, Ward DE, Camm AJ (1992). Comparison of verapamil and diltiazem in the suppression of idiopathic ventricular tachycardia. Pacing Clin Electrophysiol.

[15] Buxton A, Marchlinski F, Doherty J (1987). Hazards of intravenous verapamil for sustained ventricular tachycardia. Am J Cardiol.

